# The Role of Formyl Peptide Receptor 1 in Uterine Contraction During Parturition

**DOI:** 10.3389/fphar.2021.696697

**Published:** 2021-07-29

**Authors:** Chaolu Chen, Shuaiying Zhu, Long Bai, Meihua Sui, Danqing Chen

**Affiliations:** ^1^ Department of Obstetrics and Gynecology, Women’s Hospital, School of Medicine, Zhejiang University, Hangzhou, China; ^2^ Department of Reproductive Endocrinology, Women’s Hospital, School of Medicine, Zhejiang University, Hangzhou, China; ^3^ School of Basic Medical Sciences and Women’s Hospital, School of Medicine, Zhejiang University, Hangzhou, China; ^4^ Cancer Center, Zhejiang University, Hangzhou, China

**Keywords:** parturition, myometrium, labor, transcriptome, FPR1, myometrial contraction

## Abstract

Parturition involves the transformation of the quiescent myometrium into a highly excitable and contractile state, a process that is driven by changes in myometrial gene expression. This study aimed to identify myometrial transcriptomic signatures and potential novel hub genes in parturition, which have great significance for understanding the underlying mechanisms of successful parturition and treating labor-associated pathologies such as preterm birth. In our study, comparative transcriptome analysis was carried out on human myometrial tissues collected from women undergoing caesarean section at term in the presence (TL = 8) and absence of labor (TNL = 8). A total of 582 differentially expressed genes (DEGs) between TL and TNL tissues were identified. Gene ontology (GO), Kyoto encyclopedia of genes and genomes (KEGG) and gene set enrichment analysis (GSEA) revealed that the DEGs were enriched in signal transduction, regulation of signaling receptor activity, inflammatory response, cytokine-cytokine receptor interaction, IL-17 signaling pathway, TNF signaling pathway, among others. Thus, transcriptome analysis of the myometrium during term labor revealed that labor onset was associated with an inflammatory response. Moreover, protein-protein interactions network analysis identified FPR1, CXCL8, CXCL1, BDKRB2, BDKRB1, and CXCL2 as the hub genes associated with onset of labor. Formyl peptide receptor 1 (FPR1) was highly expressed in laboring myometrial tissues, with the activation of FPR1 *in vitro* experiments resulting in increased myometrial contraction. Our findings demonstrate the novel role of FPR1 as a modulator of myometrial contraction.

## Introduction

Successful parturition depends on the transformation of the myometrium from a quiescent state to a highly excitable and contractile laboring state. The mechanisms involved in this transformation mainly include changes in calcium sensitivity, inflammation induction, cervical maturation, lower uterine segment formation and endocrine regulation (Carbillon et al., 2001; Shynlova et al., 2009; Menon et al., 2016). Contractility and excitability are the hallmarks of myometrial transformation, and involved the reorganization of the cytoskeleton of the myometrial cells, increase in expression of contractile proteins, and increase in intracellular calcium concentration (Flenady et al., 2014; Wray and Arrowsmith, 2021). Advancement in high-throughput sequencing technologies has allowed comparative transcriptome analysis between laboring and non-laboring myometrium to identify genes involved in regulation myometrial contractility and excitability. Results from these analyses demonstrate that the differentially expressed genes (DEGs) during spontaneous labor at term are mainly associated with the inflammatory cytokines, interleukins, chemokines, and contractile-associated genes (Weiner et al., 2010; Lui et al., 2018; Stanfield et al., 2019a; Stanfield et al., 2019b). However, most of these studies lacked comprehensive network analysis of transcriptomic data and subsequent experiments aimed at identifying key genes involved in upstream regulation of labor and their roles in induction of term labor. Additionally, the studies were ethnically homogenous, and most of the participants were African-American ethnicity (Stanfield et al., 2019b). More gene expression profiling information in other ethnicities would be an addition to our existing knowledge base of parturition. Motivated by these considerations, we compared RNA expression profiles between laboring and non-laboring myometrium from Asian Chinese ethnicity to identify DEGs and functional pathways involved in parturition. We also constructed PPI networks to identify hub gene associated with labor onset and carried out our further analysis to explore its molecular mechanisms in the onset of labor. Through *in vitro* experiments, we found that Formyl peptide receptor 1 (FPR1) was highly expressed in laboring myometrium and was identified as a novel core gene in the regulation of myometrial contraction and onset of labor.

FPR1, a G-protein-coupled receptor, is mainly expressed in neutrophils and monocytes and plays a role in triggering G protein-induced signaling cascades leading to chemotaxis, phagocytosis, calcium flux, and release of pro-inflammatory mediators (McDonald et al., 2010; Wenceslau et al., 2016). Recent studies have reported that activation of FPR1 by binding with its chemotactic peptide ligand N-Formyl-Met-Leu-Phe (fMLF), promoted migration of neutrophil and vascular smooth muscle cells (Wenceslau et al., 2016; Wenceslau et al., 2019). However, there have not been any studies about the expression of FPR1 in myometrium and its function in myometrial cells. In general, we firstly explored the roles of FPR1 activation in myometrial contraction. Findings from this study give an in depth understanding of the molecular mechanisms underlying the activation of the myometrium to initiate term labor.

## Materials and Methods

### Sample Collection

This study was approved by the Ethics Committee of Women’s Hospital, School of Medicine, Zhejiang University. All participants provided written informed consent. The myometrial tissues were excised from the upper margin of the lower uterine segment incision during caesarean section. The tissues were snap-frozen in liquid nitrogen and preserved at −80°C until further analysis. Myometrial tissues were collected from women undergoing caesarean section at term (37 weeks 0 day–41 weeks and 6 days) in the absence of labor (TNL, *n* = 8) and with evident onset of labor (TL, *n* = 8). Labor was defined as the occurrence of regular uterine contractions followed by cervical effacement or dilation. The indications for C-section with TNL were abnormal fetal position or previous uterine surgery, while the indications for TL were arrested labor or fetal distress. Exclusion criteria included maternal uterine malformation, multiple pregnancies, cervical cerclage, fetal aneuploidy or lethal fetal abnormalities and serious maternal medical conditions. Demographic and clinical characteristics of the participants are described in Table 1.

**TABLE 1:** Demographic and clinical characteristics of recruited pregnant women.

	Term labor (*n* = 8)	Term not in labor (*n* = 8)	*p* value
Maternal age, years, median (IQR)	31 (28–33)	31.5 (29–36)	0.491
Body mass index, kg/m^2^ (IQR)	25.7 (25.2–28.9)	25.4 (24.0–27.4)	0.318
Gestational age at delivery, weeks (IQR)	39.2 (38.2–40.0)	38.5 (38.0–38.7)	0.102
Birth weight, g (IQR)	3,560 (3,015–3,700)	3,185 (2,990–3,283)	0.127

Data presented as median (interquartile range) and analyzed by Mann-Whitney *U* test. *p* > 0.05 showed no significant difference between two groups.

### RNA Sequencing

Total RNA was isolated from myometrial tissues using TRIzol reagent (Invitrogen, Carlsbad, CA, United States) following the manufacturer’s procedure. The RNA amount and purity of each sample was quantified using NanoDrop ND-1000 (NanoDrop, Wilmington, DE, United States). The RNA integrity was assessed by Agilent 2,100 with RIN number >7.0. A cDNA library constructed by technology from the pooled RNA from 16 samples of myometrium was sequenced in Illumina NovaseqTM 6,000 platform (LC Sciences, Hangzhou, China) following the recommended protocol.

### Transcriptome Profiles Analysis

First, Cutadapt was utilized to eliminate the reads with adaptor sequences, reads with poor quality, and undetermined reads. Subsequently, the pre-processed valid data were mapped to the *Homo sapiens* reference genome using Hisat software. StringTie was used to assemble transcripts and estimate mRNA expression levels by calculating Fragments/kb of exon model per Million mapped reads (FPKM). Using R package-edgeR, we selected DEGs and mRNAs with log2 fold change>1 or log2 fold change <−1 (*p* value < 0.05). We performed Gene Ontology (GO) and Kyoto Encyclopedia of Genes and Genomes (KEGG) pathway analyses to identify the molecular functions, cellular components, biological processes and signaling pathways enriched by DEGs.

### Gene Set Enrichment Analysis

GSEA is a computational method that sequence the gene according to the expression level of diverse biological processes and determines if the gene sets defined initially are statistically significant. In our study, the data was analyzed in GSEA software (version 4.0.3), and “h.all.v7.1. symbols.gmt” from Molecular Signatures Database (MSigDB) was used to run with 1,000 permutations. All other parameters were set to default. The gene sets showing false discovery rates (FDR) < 0.25 were considered enriched.

### Protein-Protein Interaction Network Analysis

The online STRING tool (http://www.string-db.org/) was employed to explore the interactions between DEGs, which was selected with the interaction score (highest confidence) >0.9, and the protein-protein network was visualized through Cytoscape software (version 3.7.2). Then we used the Cytohubba plugin to identify the hub genes by overlapping the top 30 genes based on 12 algorithms. Molecular Complex Detection (MCODE), another plugin of Cytoscape, was utilized to screen the entire PPI network’ core modules with default parameters (Degree cut-off = 2, Node score cut-off = 0.2, K-core = 2 and Max depth = 100).

### Cell Culture and Treatment

Human primary uterine myometrial cells (HutSMCs) were obtained from ATCC. Cells were maintained in Vascular Cell Basal Medium (PCS-100-030, ATCC) supplemented with Vascular Smooth Muscle Cell Growth Kit (PCS-100-042, ATCC) and cultured in an incubator with 5% CO_2_ at 37°C. All the experiments were performed on myometrial cells at the third to the sixth passage. The K-562 (human myelogenous leukemia cell line) cells were grown in RPMI 1640 medium (Life Technologies) supplemented with 10% FBS.

N-Formyl-Met-Leu-Phe (fMLF, agonist of FPR1, MCE, United States) and Boc-MLF TFA (tBOC, antagonist of FPR1, MCE, United States) were dissolved in dimethyl sulfoxide (DMSO, Sigma, United States) to a final concentration of less than 0.1%. After reaching 80% confluence, HutSMCs were serum-starved for 12 h followed by treatment with DMSO, fMLF (10 μM), tBOC (5 μM) or a combination of fMLF and tBOC for 24 h. The concentrations of these two reagents are non-toxic dose to cells, and its election was based on preliminary experiments (Supplementary Figure 1).

### Reverse Transcription and Quantitative Real-Time PCR

The RNAiso Plus reagent (Takara Bio, Japan) was employed to extract total RNA from myometrial tissues and cells as per the manufacturer’s protocol. Next, the RNA was reverse transcribed to cDNA using PrimeScript™ RT reagent Kit containing gDNA Eraser (Takara Bio, Japan). We performed RT-qPCR in a 7900HT Sequence Detection System (Applied Biosystems, Foster City, CA) using SYBR Premix Ex Taq™ kit (Takara Bio, Japan). The PCR program involved 40 cycles of 95°C for 30 s, 95°C for 5 s, and 60°C for 30 s. Specific primers used for PCR amplification were synthesized by Sangon (Shanghai, China) with the following sequences: GAPDH (a housekeeping gene),

5′-TCA​GTG​GTG​GAC​CTG​AC-3′ (forward) and

5′-TGC​TGT​AGC​CAA​ATT​CGT​T-3′ (reverse); FPR1,

5′-TGG​GAG​GAC​ATT​GGC​CTT​TC-3′ (forward) and

5′-GGA​TGC​AGG​ACG​CAA​ACA​C-3′ (reverse); COX2,

5′-TAA​GTG​CGA​TTG​TAC​CCG​GAC-3′ (forward) and

5′-TTT​GTA​GCC​ATA​GTC​AGC​ATT​GT-3′ (reverse); CX43,

5′-TGG​TAA​GGT​GAA​AAT​GCG​AGG-3′ (forward) and

5′-GCA​CTC​AAG​CTG​AAT​CCA​TAG​AT-3′ (reverse).

Each sample was analyzed in triplicate and the average value used to calculate cyclic threshold (Ct) value. The 2^−ΔΔCt^ method was used to determine the relative expression levels of target genes.

### Protein Extraction and Western Blot Analysis

Protein extraction was carried out by lysing myometrial tissues or cells for 30 min at 4°C using RIPA lysis buffer followed by centrifugation (14,000 rpm; 15 min) at 4°C. Protein concentration was determined using BCA assay kit (Thermo Scientific, United States). Equal protein samples were resolved on a 12% sodium dodecyl sulfate polyacrylamide gel (SDS-PAGE), then transferred onto polyvinylidene fluoride membranes (Millipore, Billerica, MA, United States). Blocking was done for 1 h at room temperature (RT) using 5% bovine serum albumin (BSA, Sigma-Aldrich, United States), followed by incubation with primary antibodies (1:500 dilution for FPR1, ab113531, Abcam; 1:1,000 dilution for OXTR, Proteintech, α-SMA, ab5694, Abcam, pMLC, #3671, CX43, #3512, p-ERK, #4370, p-JNK, #4668, p-p38, #4511, CST; 1:2000 dilution for GAPDH and β-tubulin, Abcam) overnight at 4°C. Thereafter, the membranes were incubated with horseradish peroxidase-conjugated secondary antibodies (1:10,000, Abcam) for 1 h at RT. Signal detection was achieved using enhanced chemiluminescence detection reagent (Sigma-Aldrich). Band intensities were evaluated using ImageJ software and normalized using GAPDH or β-tubulin expression.

### Immunofluorescence Analysis

The myometrial tissues were sliced into 5 μm-thick frozen sections, which were then incubated with a primary rabbit antibody against FPR1 (1:200, ab113531, Abcam) and a mouse anti-myeloperoxidase (MPO) antibody (1:200, 66177-1-Ig, Proteintech) overnight at 4°C. Afterwards, the section were washed with PBS, and then treated with the secondary antibodies, Alex Fluor^®^488-conjugated anti-rabbit antibody (1:400, SA00006-2, Proteintech) and CY3-conjugated anti-mouse antibody (1:300, SA00009-1, Proteintech) in the dark at RT for 1 h. Nuclei-staining was done by incubating sections in 5 μg/ml 4,6-diamidino-2-phenylindole (DAPI, Sigma) for 10 min, and then the sections were mounted on glass slides for immunofluorescence analysis.

We cultured the myometrial cells on microscope slides placed in the 12-well plates, then fixed them in 4% formaldehyde for 1 h, permeabilized using 0.2% Triton X-100 and then blocked with 1% BSA for 1 h. The cells were then incubated with a primary rabbit antibody against FPR1 (1:200, ab113531, Abcam) or pMLC (1:100, #3671, CST) overnight. Thereafter, the cells were incubated with FITC fluorescently labeled secondary antibody (1:60, SA00003-2, Proteintech) at RT for 1 h. Cell nuclei were labeled with DAPI. Fluorescent images were captured using a confocal fluorescent microscope.

### Transmission Electron Microscopy

We performed transmission electron microscopy (TEM) as described previously (Chen et al., 2019). First, HutSMCs (5 × 10^6^) were digested with trypsin, centrifuged (1,000 rpm; 5 min), and the supernatant discarded. The cells were then rinsed with PBS, fixed in 2.5% glutaraldehyde at 4°C for >4 h, followed by incubation with 1% osmic acid for 1 h at RT. The samples were dehydrated using 50, 70, 90, 100% ethanol solution and 100% acetone solution for 15 min at RT, embedded for 4 h and then cut into 70 nm ultrathin sections. The sections were placed on copper grids and dyed with uranyl acetate and lead citrate before analysis. We used a transmission electron microscope (Tecnai G2 Spirit 120kV, Thermo FEI, United States) to observe the ultrastructural morphology.

### Cell Contraction Assay

A collagen gel contraction assay was performed as previously described (Boyle et al., 2019). This assay was applied to evaluate the impact of FPR1 activation on the spontaneous contraction of myometrial cells. In brief, we re-suspended the myometrial cells (10^5^ cells/well) in type I rat tail collagen (Gibco). The mixture was transferred to 12-well cell culture plates, incubated in 37°C to allow collagen polymerization for 30 min and then treated with fMLF (10 μM) or tBOC (5 μM). The collagen was gently detached from the well, incubated overnight at 37°C and the wells were photographed at 0 and 24 h using a scanner (Leica, Germany). We utilized the ImageJ software to measure the gel areas. The gel area measurements at 24 h were calculated as a proportion of the mean gel areas at the 0 h time point for each separate experiment.

### Calcium Imaging

Myometrial cells (7,000/well) were seeded in 8-well µ-Slide (ibidi, Germany) imaging chambers and incubated overnight. The following day, the cells were washed, loaded with 5 μM Fluo-4 AM (Beyotime, China) Ca^2+^ indicator and incubated at 37°C for 30 min. Thereafter, the loaded cells were rinsed and further incubated in the dark for an additional 30 min at RT for Fluo-4 AM ester hydrolysis to occur. The cells were imaged before and after stimulation with oxytocin (100 nM) for 60 s, with images being acquired every 10 s for 130 s using a confocal microscopy.

### Statistical Analysis

The statistical analysis method of RNA-seq data has been introduced separately previously in method part. Experimental data are shown as means ± SD of three separate experiments. All data analyses were completed using the SPSS 12.0 statistical software (SPSS, Inc., Chicago, IL). Differences in paired data were analyzed using the parametric student *t*-test. Multiple treatments were analyzed using one-way ANOVA and means separated by Tukey’s tests. *p* value of <0.05 defined statistical significance.

## Results

### Identification and Enrichment Analysis of DEGs Between Laboring and Non-Laboring Myometrium

**FIGURE 1:**
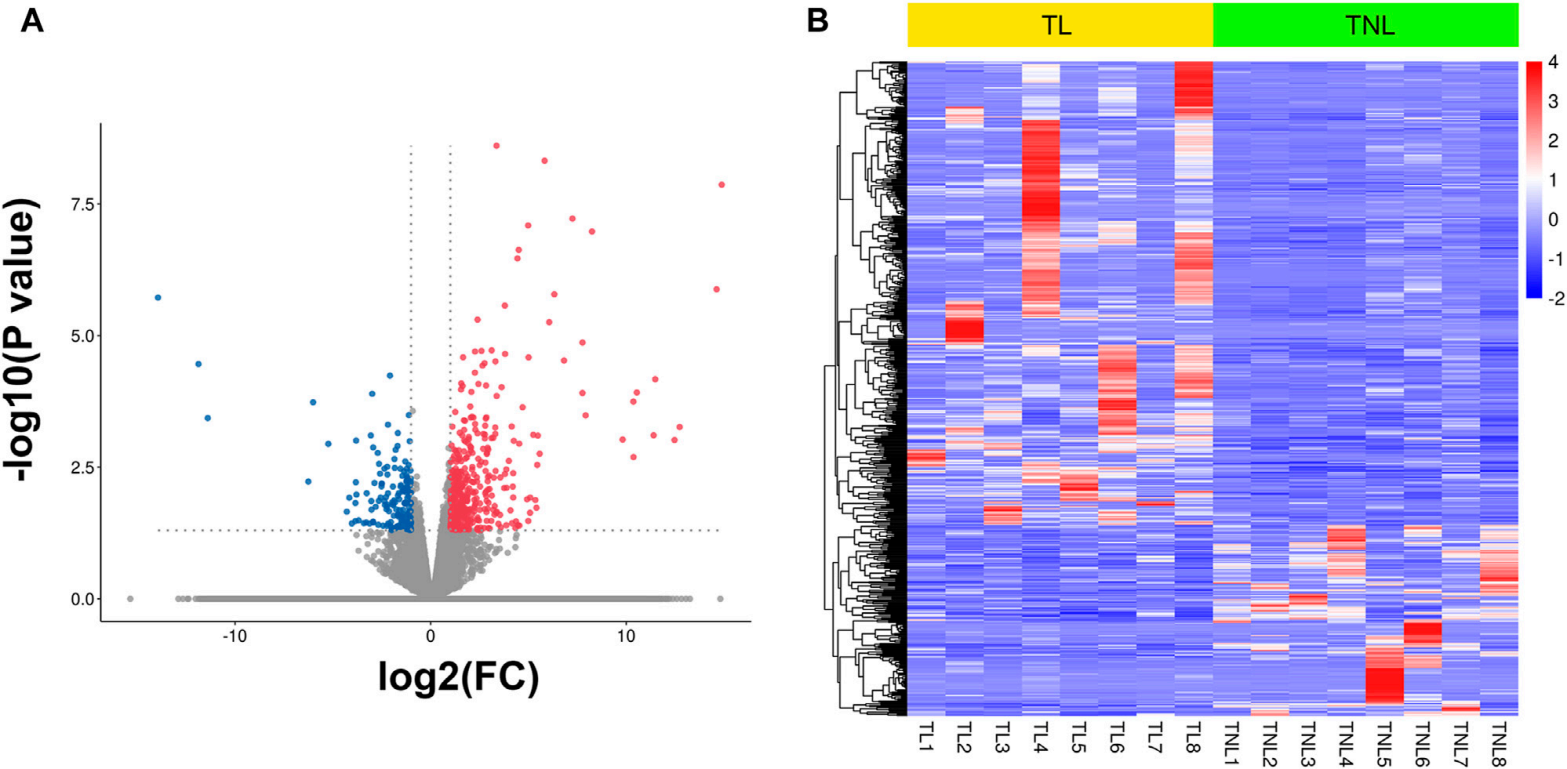
Myometrial transcriptional signatures at spontaneous human term labor (TL) and at term not in labor (TNL). **(A)** Volcano plot of distribution trends for differentially expressed genes (DEGs) between TNL and TL. Up-regulated genes are presented as red dots and down-regulated are blue. **(B)** Heatmap of expression levels for all DEGs between TL and TNL myometrium. High expression levels are shown in red, while low expression levels are in blue. Color key equals log2 expression levels.

To generate myometrial transcriptomic signatures of human term labor (TL) and at term not in labor (TNL), we used the cut-off point of |log2 fold change|≥1 and *p* value < 0.05. A total of 582 DEGs were identified, of which 412 were up-regulated while 170 were down-regulated (Figure 1A, Supplementary Table 1). Clustering analysis was conducted using the heatmap package in R language (version 1.0.12) (Figure 1B).

**FIGURE 2:**
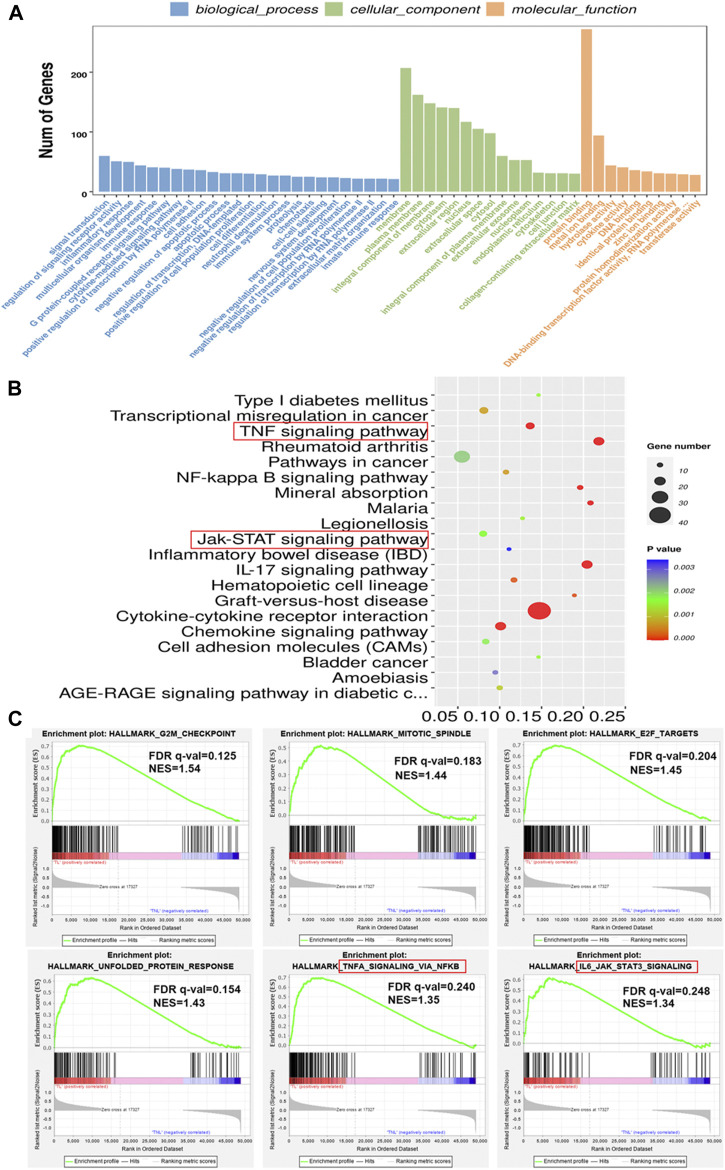
Enrichment analysis of DEGs in laboring and non-laboring myometrium. **(A)** GO annotations of DEGs involved in three categories: biological process (BP), cellular component (CC) and molecular function (MF). **(B)** Bubble plot of enriched pathways by KEGG pathway analysis between TL vs. TNL group. *Y* axis represents KEGG pathway, *X* axis rich factor represents the percentage of DEGs enriched in pathway. *p* value <0.05 was used as thresholds in selecting the 20 most significant KEGG pathway **(C)** Gene set enrichment analysis using Hallmark gene sets from MSigDB. NES: normalized enrichment score.

GO and KEGG analysis were used to predict the functions and the pathways associated with the DEGs. GO annotations revealed that in terms of biological process, the DEGs were enriched in signal transduction, regulation of signaling receptor activity and inflammatory response. For cellular component, the DEGs were enriched in membrane, cytoplasm and extracellular region. In terms of molecular function, the DEGs were involved in binding processes, hydrolase activity and cytokine activity (Figure 2A). KEGG pathway analysis revealed that the 582 DEGs were significantly enriched in 42 pathways, including cytokine-cytokine receptor interaction, IL-17 signaling pathway, TNF signaling pathway, mineral absorption and chemokine signaling pathway, among others (Figure 2B). To further investigate the possible biological functions of the DEGs implicated in the process of labor onset, gene set enrichment analysis was performed using “Hallmark gene sets” of MSigDB. The results showed significant differences in the enrichment of “G2M CHECKPOINT”, “MITOTIC SPINDLE”, “E2F TARGETS”, “UNFOLDED PROTEIN RESPONSE”, “TNFA SIGNALING VIA NFKB” and “IL-6 JAK STAT3 SIGNALING” (Figure 2C). Although different bioinformatics tools were used, the results of GSEA were consistent with the results of KEGG analysis (as shown in the red rectangle in Figures 2B,C). The NES (normalized enrichment score) values revealed that expression of genes associated with proliferation, cellular stress response and inflammatory response were positively correlated with labor onset.

### Selection and Validation of Hub Genes

**FIGURE 3:**
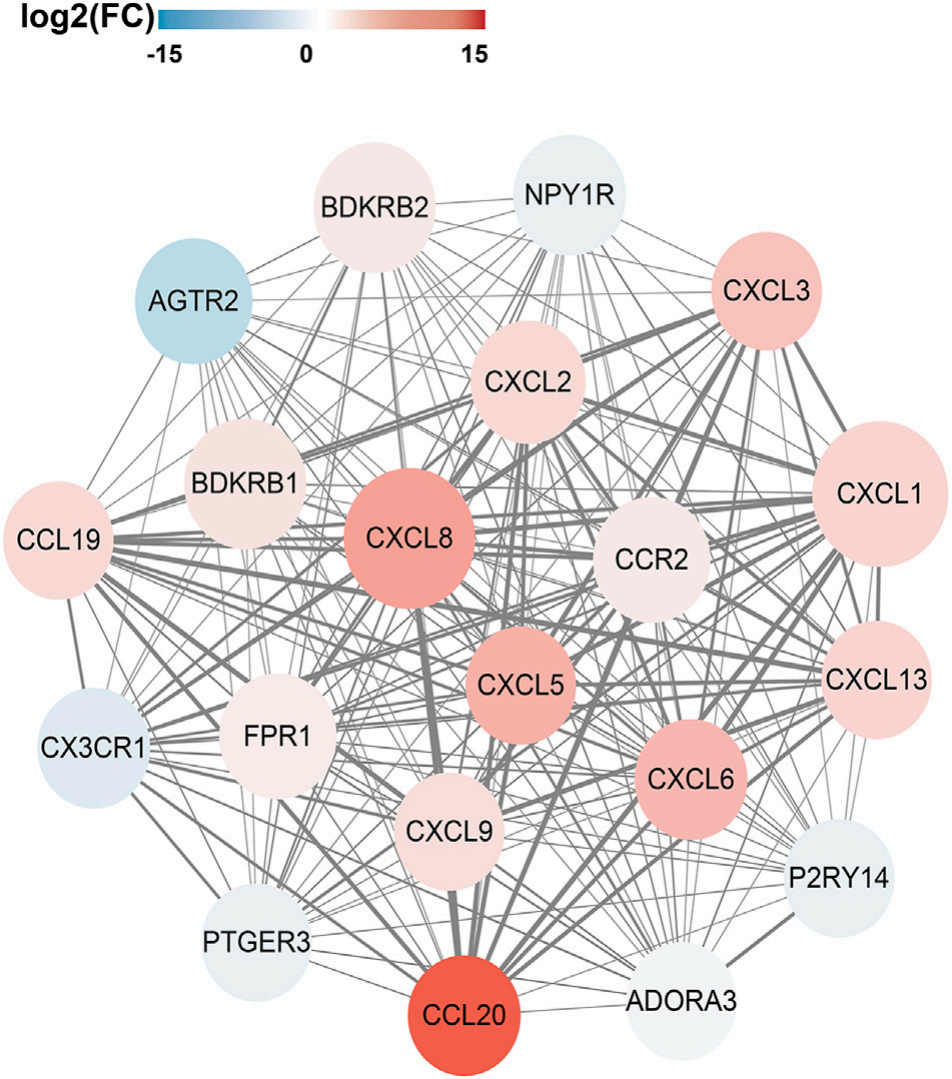
PPI network and Hub genes selection. The Protein-Protein interaction network of DEGs was constructed by Cytoscape. The red nodes represent up-regulated genes, while the blue represent down-regulated genes. The color of node varies from fold change of DEGs. Size of the node is displayed upon degree of connectivity of the node. The width of the edge represents the value of combined score from 0.9 to 1.0. The significant clusters identified from PPI network by MCODE plugin with the MCODE score >5 and number of nodes >10. The Cluster one consists of 20 nodes and 190 edges (score: 20.000).

In order to analyze the interactions between DEGs and select hub genes in parturition, we used 12 algorithms of the CytoHubba plugin in Cytoscape to rank the genes that were highly correlated with labor onset. We then selected hub genes by overlapping the top 30 ranked genes obtained using each algorithm. The six most overlapped genes were FPR1, CXCL8, CXCL1, BDKRB2, BDKRB1 and CXCL2. The top 30 ranked nodes of each algorithm were listed in Supplementary Table 2. As shown in Supplementary Figure 2, we used the MCODE (MCODE score >5 and number of nodes >10) plugin of Cytoscape to identify highly interconnected genes. From this analysis, three major clusters were identified. Among these clusters, the cluster one had the highest score (score: 20.000), contained 20 nodes and 190 edges, and matched the most hub genes (Figure 3). Functional and KEGG pathway enrichment analysis revealed that genes in this module were mainly associated with G protein-coupled receptor signaling pathway, inflammatory response, chemokine signaling pathway and cytokine-cytokine receptor interaction (Table 2). Previous studies have shown that the CXCL family, BDKRB1 and BDKRB2 promote the initiation of labor (Abbas et al., 1996; Gomez-Lopez et al., 2013; Willets et al., 2015), but the role of FPR1 in labor onset has not been studied. Thus we sought to identify the role of FPR1 gene in labor onset.

**TABLE 2:** Function and pathway enrichment analysis of genes in cluster one.

Term	Description	Count	*p* value
GO:0007186	G protein-coupled receptor signaling pathway	18	5.27E-23
GO:0006954	Inflammatory response	15	1.19E-23
GO:0007165	Signal transduction	14	9.76E-12
GO:0006935	Chemotaxis	13	3.05E-24
GO:0070098	Chemokine-mediated signaling pathway	12	3.24E-26
GO:0006955	Immune response	12	8.65E-16
GO:0005515	Protein binding	12	0.221,718
GO:0008009	Chemokine activity	10	1.45E-22
GO:0030593	Neutrophil chemotaxis	10	3.72E-20
GO:0005125	Cytokine activity	10	9.39E-16
GO:0010469	Regulation of signaling receptor activity	10	1.26E-12
GO:0005615	Extracellular space	10	9.13E-07
GO:0005576	Extracellular region	10	1.24E-05
hsa04062	Chemokine signaling pathway	12	5.98E-16
hsa04060	Cytokine-cytokine receptor interaction	12	1.24E-13
hsa04657	IL-17 signaling pathway	7	9.27E-10

### Up-Regulated Expression of FPR1 in Laboring Myometrium

**FIGURE 4:**
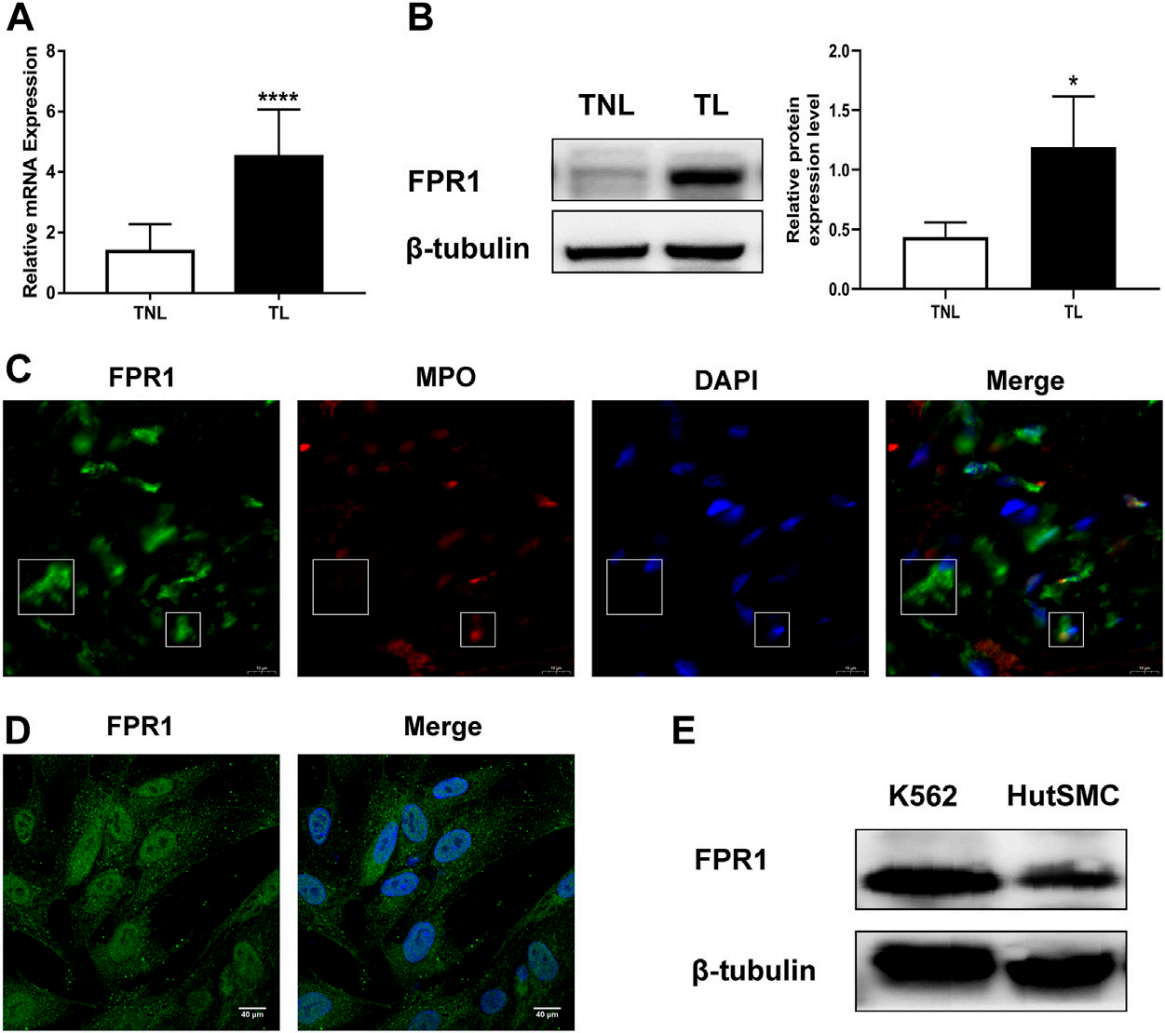
FPR1 expression in human laboring myometrium. **(A,B)** RT-qPCR and western blotting of the expression level of FPR1 in TL myometrium compared to TNL myometrium. *p < 0.05, ****p < 0.0001. **(C)** Frozen sections of TL myometrial tissues were stained by immunofluorescence for the expression of FPR1 (Alex Fluor^®^488, green) and MPO (Cy3, red), as described in Materials and Methods. Cell nuclei were stained with DAPI (blue). Images shown are representative of three independent experiments with similar results. Scale bar, 10 µm. **(D)** Immunofluorescence for the expression of FPR1 (green), co-labeled with DAPI (blue) in HutSMCs (human uterine smooth muscle cells). Scale bar, 40 µm. **(E)** The expression of FPR1 in HutSMCs with K-562 cell line as positive control by WB assay.

To evaluate the expression of FPR1 in myometrium, PCR and WB were used in laboring and non-laboring myometrium. The results found that FPR1 expression was higher in TL myometrium compared to TNL myometrium (Figures 4A,B). Since FPR1 was highly expressed in TL myometrium, we further investigated the cellular localization and distribution of FPR1 in myometrial tissues using immunofluorecence staining. As shown in Figure 4C, FPR1 was expressed by both myometrial cells (MPO negative) and tissue-infiltrating myeloid cells (MPO positive) in TL myometrium tissues. These findings implied that the expression of FPR1 by myometrial and infiltrating myeloid cells resulted in the up-regulation of FPR1 in TL myometrial tissues. In addition, cell immunofluorescence and WB analysis revealed that FPR1 was also expressed in HutSMCs in which the K-562 cell line acted as a positive control (Figures 4D,E). Thus, we used HutSMCs in subsequent cell experiments to study the function of FPR1 in myometrial contraction.

### Effect of FPR1 Activation on the Ultrastructure of HutSMCs

**FIGURE 5:**
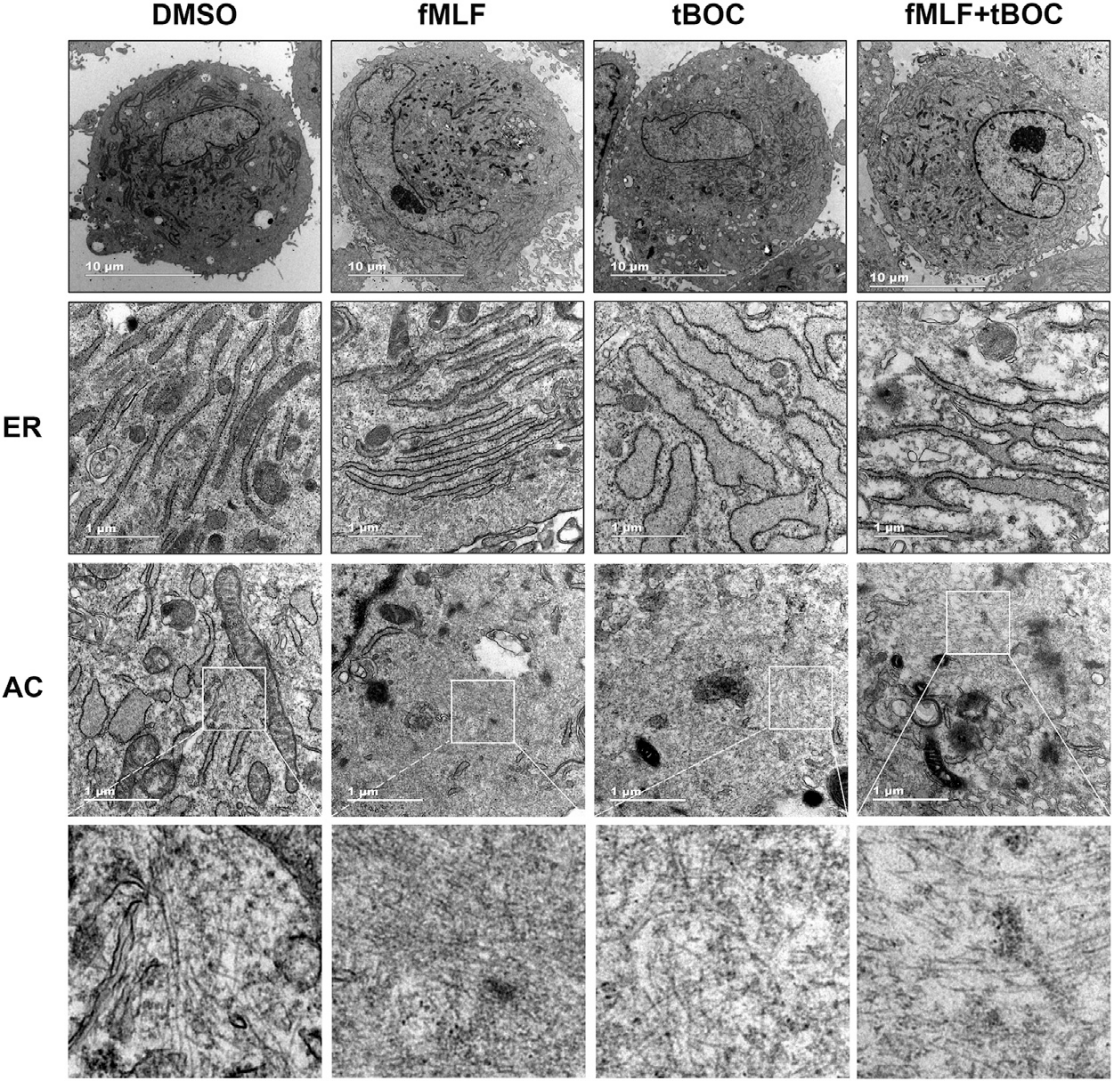
Representative transmission electron microscopy of HutSMCs with and without treatment with fMLF or tBOC. Increasing levels of magnification are indicated by scale bars, 10 and 1 μm. ER: endoplasmic reticulum; AC: actin filaments.

Given that cell contraction involves changes in cell cytoskeleton, we questioned if activation of FPR1 would alter the ultrastructure of HutSMCs to a contractile phenotype. To answer this question, we observed myometrial cells after FPR1 agonist or antagonist treatment using a transmission electron microscope. As shown in Figure 5, we found that there was accumulation of linear endoplasmic reticulum (ER) and oval-like mitochondria in HutSMCs treated with fMLF (FPR1 agonist). In these cells, the ERs were packed tightly together in an orderly manner and the ER lumen was normal in size. The cells were rich in actin filaments (AC), which were aggregated in bundles and arranged parallel to each other throughout the cytoplasm. In the tBOC (FPR1 antagonist) group, there was accumulation of nonlinear, swollen and fragmented ERs, which appeared as a convoluted tangle of cisternae. The ER lumen was dramatically enlarged and there was an increase in the distance between each lumen. The cells appeared spherical, filopodia around the cell membrane were scarce, and actin filaments were few, scattered and disorganized. In the fMLF + tBOC group, the ERs were slightly enlarged and actin filaments were scarce.

### Effect of FPR1 Activation on the Contraction of HutSMCs

**FIGURE 6:**
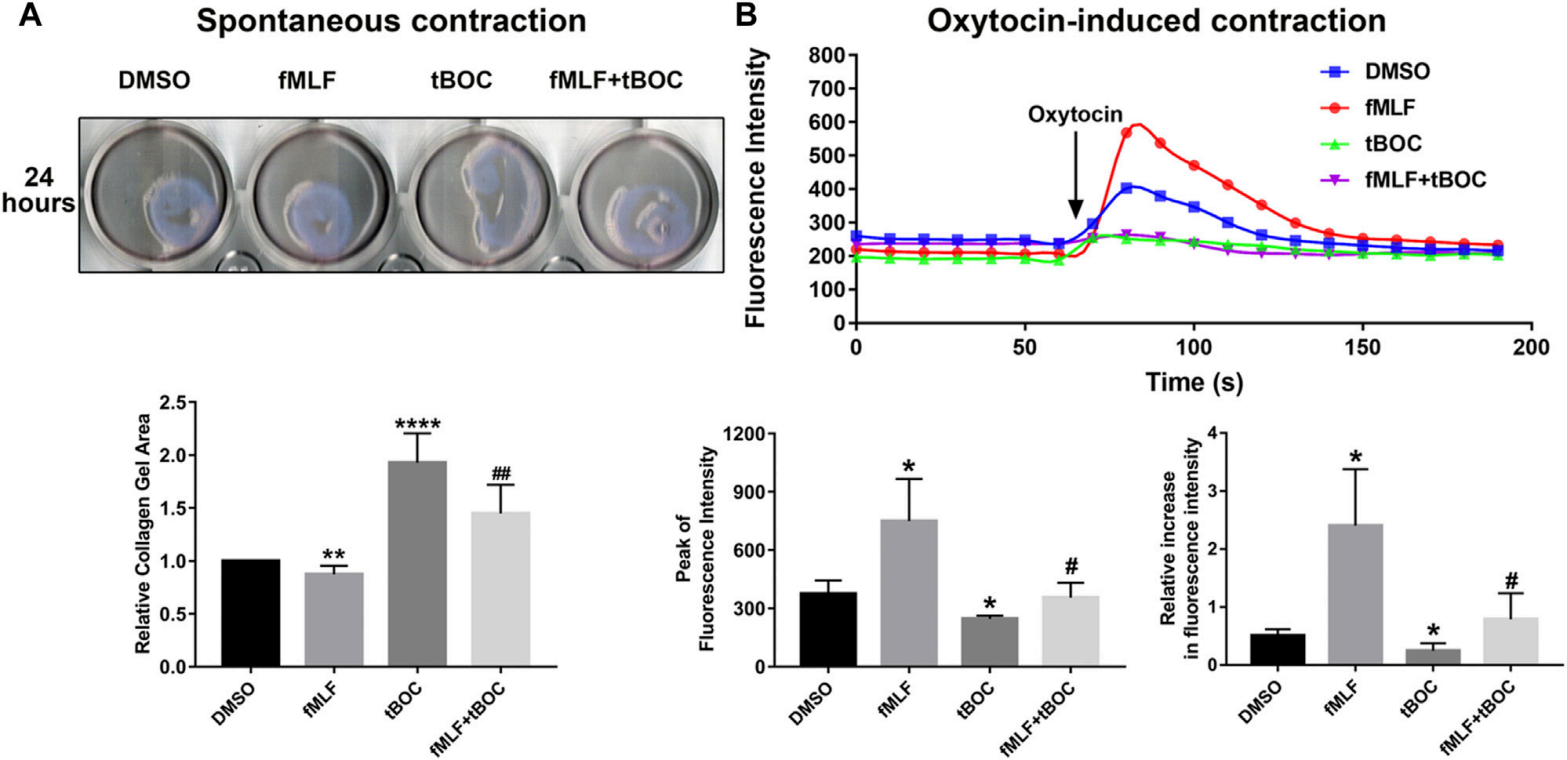
FPR1 activation promotes HutSMC contraction. **(A)** The effect of FPR1 on spontaneous contraction in HutSMCs treated with DMSO (control), tBOC (5 μM), fMLF (10 μM), or fMLF/tBOC combination for 24 h, which was determined by gel areas. **(B)** The effect of FPR1 on oxytocin-induced contraction in HutSMCs treated with DMSO (control), tBOC (5 μM), fMLF (10 μM), or fMLF/tBOC combination for 24 h, which was determined by intracellular calcium concentration. The concentration was reflected by fluorescence intensity. *p < 0.05 compared with control, ^#^ *p* < 0.05 compared with fMLF group.

Contraction of uterine myometrial cells can be due to spontaneous contraction or oxytocin-induced contraction. To investigate the role of FPR1 activation in the contraction of myometrial cells, we evaluated spontaneous cell contractility by measuring the gel area in the cell contraction assay and examined oxytocin-induced contraction by calcium imaging. The results of the contraction assay revealed that HutSMCs treated with fMLF exhibited smaller gel areas than DMSO treated HutSMCs, indicating that fMLF positively regulated spontaneous cell contraction. HutSMCs treated with fMLF and tBOC showed larger gel areas than the fMLF group, indicating tBOC abrogated the effect of fMLF on cell contraction (Figure 6A). As shown in Figure 6B, fMLF induced a rapid and transient increase in calcium flux in HutSMCs, which was significantly diminished by tBOC. Cells treated with fMLF were higher in the peak of calcium concentration and relative increase of calcium concentration, which were inhibited by tBOC.

### Effect of FPR1 Activation on Contraction-Associated Proteins and MAPKs Phosphorylation

**FIGURE 7:**
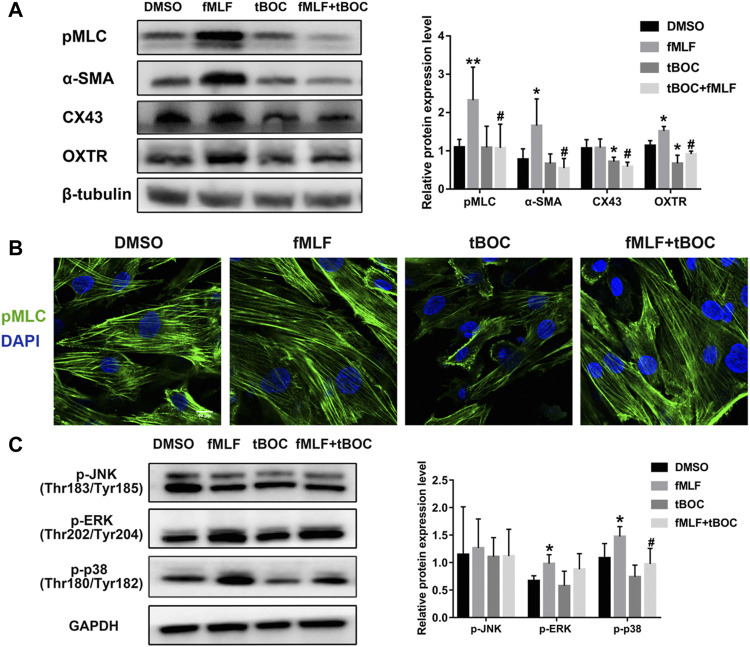
FPR1 activation promoted expression of contractile proteins and MAPKs phosphorylation. **(A)** The protein expression of contractile proteinss in HutSMCs treated with DMSO (control), tBOC (5 μM), fMLF (10 μM), or fMLF/tBOC combination. **(B)** Distribution and expression of MLC phosphorylation in HutSMCs. Scale Bar = 40 μm. **(C)** The effect of FPR1 on MAPKs phosphorylation in HutSMCs treated with DMSO (control), tBOC (5 μM), fMLF (10 μM), or fMLF/tBOC combination for 24 h *p < 0.05 compared with control, ^#^ *p* < 0.05 compared with fMLF group.

In order to investigate the role of FPR1 activation in the expression of CAPs (contraction-associated proteins), which are downstream effectors of contractions, WB and cell immunofluorescence were used. As shown in Figure 7A, the protein expression levels of pMLC (phosphorylated myosin light chain), α-SMA (alpha-smooth muscle actin) and OXTR (oxytocin receptor) were up-regulated in HutSMCs treated with fMLF, an effect that was inhibited by tBOC. However, the FPR1 agonist had little effect on CX43 (connexin 43) protein expression. We also examined phosphorylation of MLC20 using immunofluorescence and found that FPR1 activation promoted MLC20 phosphorylation (Figure 7B). Activation of the MAPKs signaling pathway is involved in regulation of myometrial contraction through the up-regulation of CAPs (Li et al., 2004). We evaluated the effect of FPR1 activation on phosphorylation of MAPKs. We found that FPR1 activation significantly promoted phosphorylation of p38 MAPK and ERK in HutSMCs, while tBOC attenuated fMLF-induced phosphorylation of p38 (Figure 7C).

## Discussion

During pregnancy, myometrial cells maintain a relaxed and compliant state. Once parturition initiate, myometrial cells respond to stimulation by increasing concentrations of contractile agonists (e.g., oxytocin, stimulatory prostaglandins) that drive the contractions required for the delivery of the fetus. The changes in myometrial cell state and function involve the remodeling of cell cytoskeleton and changes in expression of contractile proteins as well as intracellular calcium concentrations (Aguilar and Mitchell, 2010; Mendelson et al., 2019). Improper contractility and excitability of the myometrium can cause serious complications, including preterm birth (delivery earlier than 37 weeks gestation). Term labor is induced by normal physiological processes at the end of the gestation period, while preterm labor is often induced by pathological factors that cause the delivery of the fetus before the end of the gestation period (Romero et al., 2006; Keelan, 2018). In both cases, there is induction of regular and high-intensity contractions of the uterus, causing a significant increase in intrauterine pressure that results in the delivery of the fetus. Therefore, fully understanding the upstream regulatory mechanisms for the transition process of myometrium activation and finding effective genes involved in myometrial contraction may benefit the prevention of preterm birth. Our transcriptome analysis revealed that labor onset was associated with immune response, including genes/pathways involved in cytokine signaling, and chemotaxis, which was consistent with findings from previous studies (Stanfield et al., 2019a; Stanfield et al., 2019b). We newly found that unfolded protein pathway and cellular stress response participated in labor onset. Recent studies have confirmed *in vitro* experiments in which unfolded protein response was activated in the myometrium after spontaneous labor (Liong and Lappas, 2014).

In this study, we mainly focused on the role of FPR1, a gene that is highly expressed in laboring myometrium, in labor onset. As a chemoattractant receptor, FPR1 plays an important role in neutrophil directional migration by promoting increase in calcium flux and actin polymerization (McDonald et al., 2010; Wenceslau et al., 2016). Contraction of myometrial cells is mainly regulated through changes in intracellular calcium concentration and cytoskeleton remodeling. However, the role of FPR1 in activating myometrial cells to the excitable and contractile phenotype is unknown. Based on this previous knowledge, we hypothesized that activated FPR1 in the myometrium not only functions as a receptor in the immune system as previously reported in other tissues, but is also crucial for myometrial contraction through regulation of calcium flux and cytoskeleton. We demonstrated that FPR1 regulated cell contraction through the remodeling of myometrial cellular ultrastructure particularly the ER and cytoskeleton. Inhibition of FPR1 activation resulted in enlarged ER lumen, which played an important role in calcium storage (Görlach et al., 2006). Sufficient intracellular Ca^2+^ levels are necessary for myometrial cell contraction. Enlarged ERs store more calcium ions in their lumens, thereby reducing cytoplasmic Ca^2+^ concentration and consequently inhibiting cell contraction. FPR1 activation also promoted actin polymerization in myometrial cells. The ultrastructure analysis of contractile myometrial cells showed that cells had tightly packed thin filaments in the cytoplasm. The cytoskeleton is a complex, dynamic network of interlinking protein filaments present in the cytoplasm, which enable cells undergo changes in shape as well as migrate and contract. Recent studies have also found that FPR1 regulates actin polymerization to promote cell motility and contractility in other cell types, such as immune cells (Wen et al., 2019; Wenceslau et al., 2019). Contraction of cells involves the process of cytoskeleton reconstruction, which not only generates contractile forces within each cell, but also transmits contractile forces through attachment and linkage of groups of cells and interaction with tissue matrix (Koenderink and Paluch, 2018). These results demonstrate that myometrial cells have a rich cytoskeletal structure and that FPR1 agonists provoke changes in actin filaments to stimulate myometrial activation for efficient contractility. In summary, FPR1 activation remodels the cell ultrastructure to transform the cell into the contractile state, a finding that was also confirmed by cell contraction assay.

Uterine tissue can spontaneously contract due to the excitation-contraction coupling of smooth muscle, which is primarily regulated by intracellular calcium concentrations ([Ca^2+^]i). The dynamic changes in intracellular calcium concentrations are mainly regulated through the extracellular calcium pool and intracellular calcium stores (sarcoplasmic reticulum; SR). A disruption of the intracellular Ca^2+^ concentration balance is rapidly rescued by mobilization of Ca^2+^ from either the extracellular pool through the L-type VGCCs (Voltage-gated calcium channels) or release from the SR (Thorneloe and Nelson, 2005). Extracellular Ca^2+^ drives spontaneous uterine contractions by stimulating the excitation-contraction coupling of the smooth muscles. Blocking VGCCs prevents entry of extracellular calcium and significantly abolishes spontaneous uterine activity. Oxytocin is a hormone that is widely used in the clinic to induce labor, strengthen uterine contractions, or to control bleeding after childbirth. The principal mechanism of oxytocin-induced uterine contraction is dependent on the activation of L-type VGCCs and OXTRs (oxytocin receptors). We found that FPR1 increased the expression of OXTR in myometrial cells, indicating that FPR1 can increase cell responsiveness to oxytocin and activate OXTR. The activation of OXTRs promotes Ca^2+^ release from the SR through increased production of inositol 1, 4, 5-triphosphate catalyzed by phospholipase C (Vrachnis et al., 2011). The elevation of Ca^2+^ in the cell results in the binding of Ca^2+^ to the calcium binding protein, calmodulin (CaM), to activate the intracellular myosin light chain kinase (MLCK) and subsequently form the Ca^2+^-(CaM) 4–MLCK complex. The Ca^2+^-(CaM) 4–MLCK complex induces phosphorylation of serine residue 19 on the myosin light chain (MLC) which causes a structural change in the structure of MLC to complete uterine myocyte contraction. MLC phosphorylation facilitates the binding of the myosin cross-bridge to the actin filament which initiates contraction via the cross-bridge cycle (Word et al., 1993; Shojo and Kaneko, 2001). In our study, we found that the FPR1 agonist increased the phosphorylation of MLC, illustrating its contractile effect on the uterus. FPR1 activation can stimulate phospholipase C to convert phosphoinositol 4,5-biphosphate to inositol 1,4,5-triphosphate, which subsequently mobilizes Ca^2+^ from storage resulting in increased intracellular Ca^2+^ (Migeotte et al., 2006). In this study, we observed that FPR1 activation promoted both spontaneous and oxytocin-induced contraction through receptor-dependent and -independent stimuli. These results demonstrated that FPR1 increased intracellular Ca^2+^ through regulating the influx of extracellular Ca^2+^ and Ca^2+^ release from SR. Increased intracellular Ca^2+^ promotes MLC phosphorylation which subsequently promotes the binding of myosin to actin to enable the myocytes to contract. In the myometrium, the myocytes are connected by gap junctions. Connexin 43 (CX43), is the main component of gap junctions. CX43 promotes electrical coupling between the myocytes by rapidly inducing the synchronous transmission of electrical impulses to the entire uterine wall, which is required for uterine contractility during labor (Tabb et al., 1992). FPR1 inhibition decreased expression of CX43, which interfered with the process of electrical impulse transmitting.

CAPs are labor effector molecules that are crucial for cell contractility and maintaining myometrial contraction during labor. Our results showed that FPR1 agonist increased the expression of CAPs, including OXTR, pMLC, α-SMA and CX43. Up-regulation of contractile protein expression not only enhances responsiveness to uterotonins such as oxytocin and PGs, but it also strengthens cell coupling by increasing gap junctions to transform the HutSMCs to the contractile state. Some studies have revealed that the MAPKs signaling pathway plays a crucial function in labor onset through regulation of CAPs (Li et al., 2004). ERK and P38 MAPK signaling pathways promote the transcription of CAPs and the synthesis of contractile regulators, regulates intracellular calcium ion concentration, and then promotes uterine contraction in the third stage of labor (Otun et al., 2005). In this study, activation of FPR1 in HutSMCs increased phosphorylation of p38 and ERK. These results demonstrate that ERK and p38 MAPK signaling pathway could participate in the process by which FPR1 activation up-regulates CAPs to regulate myometrial cell contraction.

One shortcoming of our study was the small sample size. A larger sample size is required to validate our findings.

## Conclusion

**FIGURE 8:**
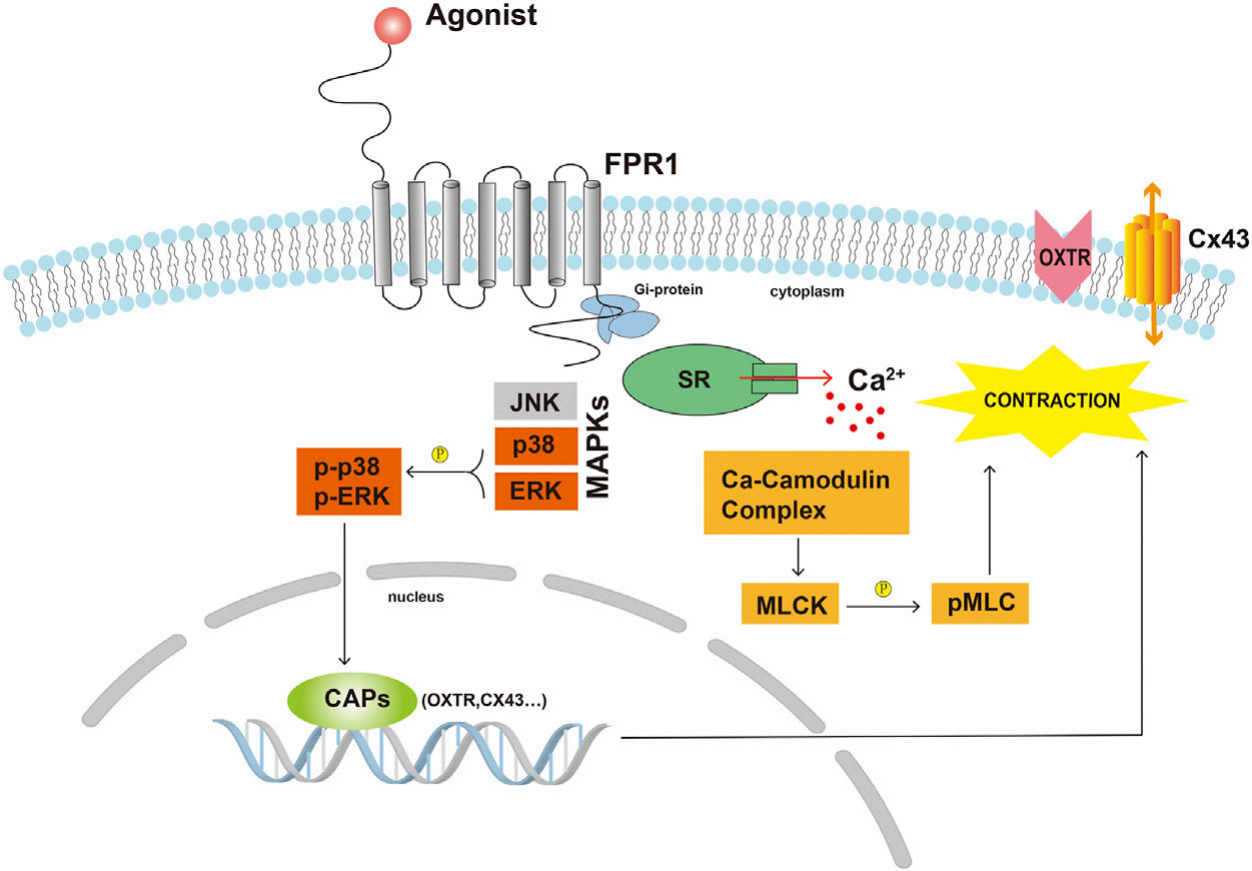
Scheme demonstrating the possible underlying mechanisms of FPR1 activation in myometrial contraction. Abbreviations: CAPs, contraction-associated proteins; Cx43, connexin 43; MLCK, myosin light chain kinase; pMLC, phosphorylated myosin light chain; OXTR, oxytocin receptor; SR, sarcoplasmic reticulum.

In conclusion, our current study explored the DEGs between TNL and TL, and demonstrated that FPR1 was highly expressed in TL myometrium. Findings from our study also revealed that activation of FPR1 promoted HutSMC contraction through regulation of pro-labor mediators, gap junction formation, intracellular Ca^2+^ and actin polymerization (Figure 8). Our findings will provide new insights into the mechanism of labor.

## Data Availability

The original contributions presented in the study are publicly available. This data can be found here: National Center for Biotechnology Information (NCBI) BioProject database under accession number GSE178781.
