# Supplementary figures and images for: The Role of Formyl Peptide Receptor 1 in Uterine Contraction During Parturition
Source: Front Pharmacol. 2021 Jul 29;12:696697. doi: 10.3389/fphar.2021.696697 (PMC8358927; doi:10.3389/fphar.2021.696697)

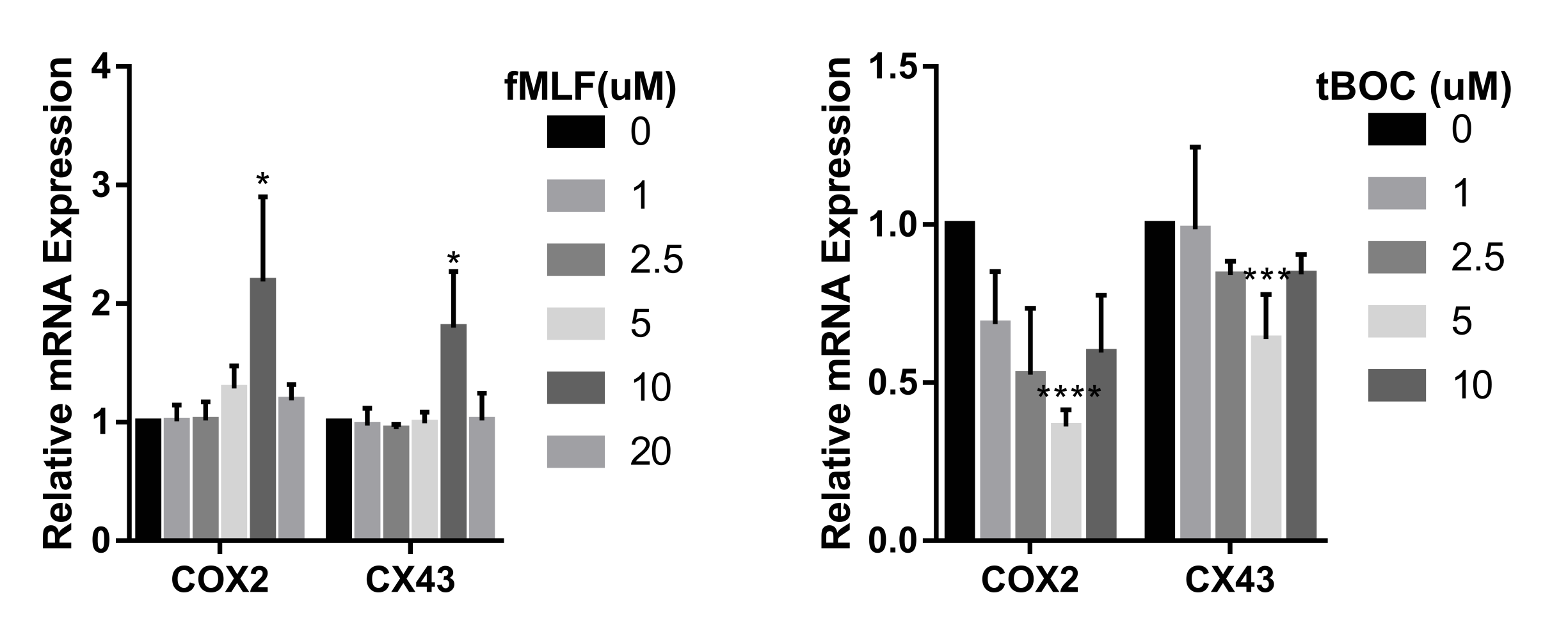

Supplement: Supplementary file 1 [file image1.tiff]

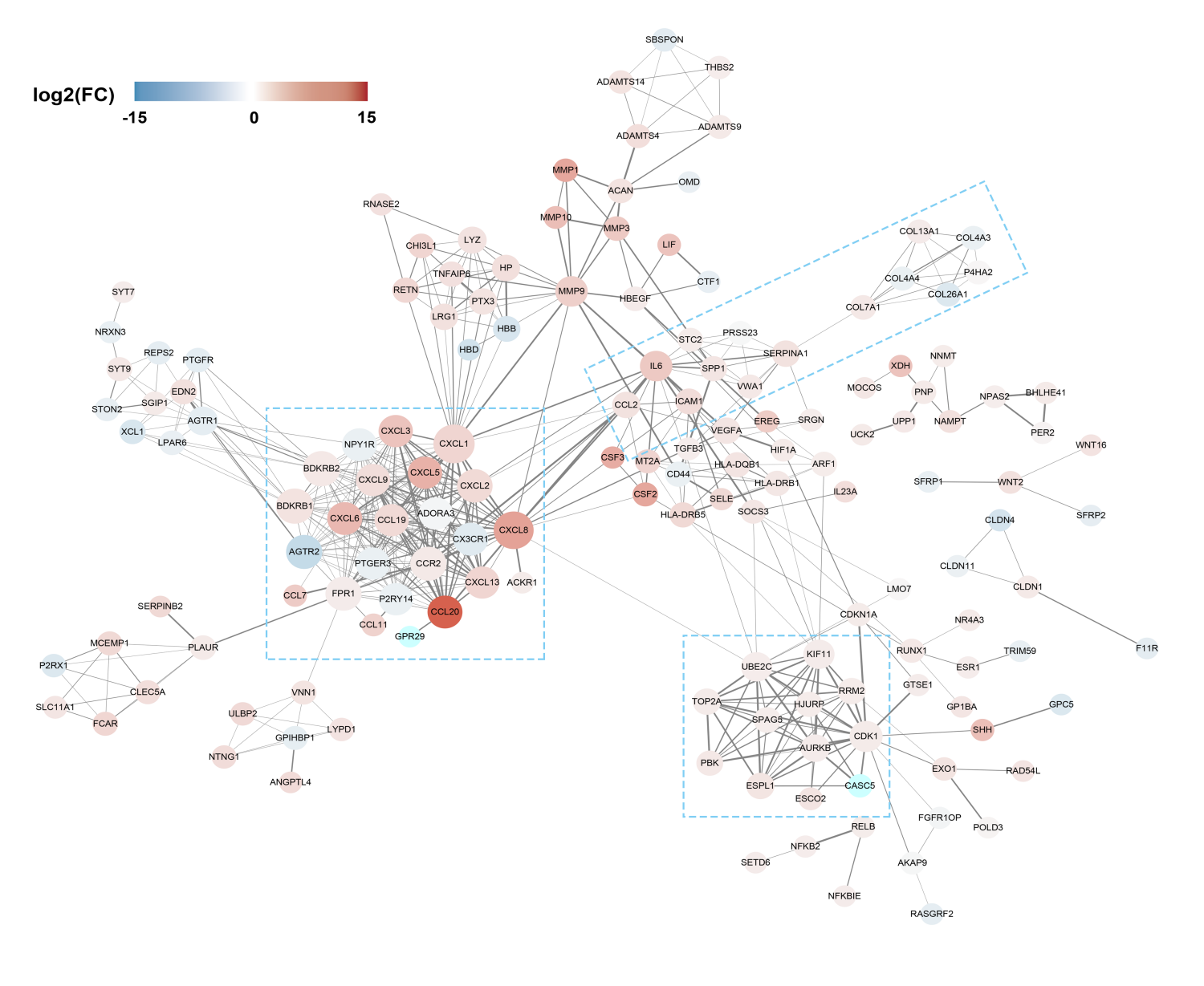

Supplement: Supplementary file 4 [file image2.tiff]
