# Supplementary material for: The Role of Formyl Peptide Receptor 1 in Uterine Contraction During Parturition
Source: Front Pharmacol. 2021 Jul 29;12:696697. doi: 10.3389/fphar.2021.696697 (PMC8358927; doi:10.3389/fphar.2021.696697)
Supplement: Supplementary file 2 [file table2.docx]

**Table S2 The hub genes analyzed by 12 topological algorithms in CytoHubba**

| **Topological algorithm** | **Top 30 genes ranked by score** |
| --- | --- |
| **Maximal Clique Centrality (MCC)** | ***CXCL1, BDKRB2, BDKRB1, CXCL8, CXCL2***, AGTR2, CCR2, CX3CR1, NPY1R, CCL19, CXCL13, CXCL6, CCL20, CXCL9, CXCL5, CXCL3, ***FPR1***, ADORA3, P2RY14, PTGER3, CDK1, KIF11, AURKB, UBE2C, TOP2A, RRM2, SPAG5, ESPL1, LYZ, HP |
| **Density of Maximum Neighborhood Component (DMNC)** | CCL19, CXCL13, CCL20, CXCL9, CXCL5, CXCL3, ADORA3, P2RY14, PTGER3, CX3CR1, NPY1R, ***FPR1***, CXCL6, ***CXCL2***, AGTR2, CCR2, ***BDKRB2, BDKRB1***, SPAG5, PBK, HBB, TNFAIP6, TOP2A, HP, PTX3, LRG1, PTGFR, CHI3L1, LPAR6, XCL1 |
| **Maximum Neighborhood Component (MNC)** | ***CXCL1, CXCL8, BDKRB2, BDKRB1***, CCR2, AGTR2, ***CXCL2***, CXCL6, ***FPR1***, CX3CR1, NPY1R, CCL19, CXCL13, CCL20, CXCL9, CXCL5, CXCL3, ADORA3, P2RY14, PTGER3, IL6, CDK1, AURKB, KIF11, UBE2C, MMP9, LYZ, CCL2, ESPL1, TOP2A |
| **Degree** | ***CXCL1, CXCL8, BDKRB2, BDKRB1***, CCR2, ***FPR1***, AGTR2, ***CXCL2***, CXCL6, CCL20, CX3CR1, NPY1R, CCL19, CXCL13 , CXCL9, CXCL5, CXCL3, ADORA3, P2RY14, PTGER3, CDK1, IL6, MMP9, KIF11, UBE2C, AGTR1, AURKB, LYZ, CCL2, ESPL1 |
| **Edge Percolated Component (EPC)** | ***CXCL8, CXCL2***, PTGER3, ***BDKRB1***, NPY1R, CX3CR1, P2RY14, ***CXCL1***, CXCL9, CXCL6, ***BDKRB2***, CCL20, ***FPR1***, AGTR2, CXCL5, CCR2, CCL19, CXCL3, ADORA3, CXCL13, IL6, MMP9, CCL2, AGTR1, LYZ, HP, LRG1, PTX3, TNFAIP6, EDN2 |
| **Bottleneck** | ***CXCL8***, IL6, ***CXCL1***, UBE2C, MMP9, ***CXCL2, FPR1***, PNP, NAMPT, ***BDKRB2***, SERPINA1, ACAN, PLAUR, AGTR1, CSF2, CDK1, VEGFA, VNN1, ICAM1, COL7A1, RUNX1, RRM2, WNT2, ARF1, HIF1A, NPAS2, SOCS3, BMP2, HBEGF, EXO1 |
| **EcCentricity** | ***CXCL8***, UBE2C, CCR2, CCL2, VEGFA, CCL19, CXCL13, IL6, MMP9, ***CXCL1***, CXCL6, CCL20, CXCL9, ***CXCL2***, ACKR1, CXCL5, CXCL3, AGTR1, ***FPR1***, CSF2, CX3CR1, ADORA3, P2RY14, ICAM1, PTGER3, AGTR2, ***BDKRB2, BDKRB1***, NPY1R, CDK1 |
| **Closeness** | ***CXCL8, CXCL1***, IL6, ***BDKRB2, BDKRB1, FPR1, CXCL2***, MMP9, AGTR2, CCR2, NPY1R, UBE2C, CXCL6, CCL20, CX3CR1, CCL19, CXCL13, CXCL9, CXCL5, CXCL3, ADORA3, P2RY14, PTGER3, VEGFA, CCL2, AGTR1, ICAM1, CDK1, KIF11, LYZ |
| **Radiality** | ***CXCL8***, IL6, ***CXCL1***, MMP9, UBE2C, ***CXCL2***, VEGFA, ***FPR1, BDKRB2, BDKRB1***, AGTR2, NPY1R, CCR2, CCL2, CXCL6, CCL20, CX3CR1, CCL19, CXCL13, CXCL9, CXCL5, CXCL 3, ADORA3, P2RY14, PTGER3, ICAM1, CSF2, AGTR1, SOCS3, SPP1 |
| **Betweenness** | ***CXCL8***, UBE2C, MMP9, IL6, ***FPR1, CXCL1***, CDK1, AGTR1, SERPINA1, ACAN, PLAUR, VNN1, COL7A1, VEGFA, RUNX1, CSF2, SOCS3, HBEGF, ICAM1, EXO1, SYT9, KIF11, ***BDKRB2, BDKRB1***, ADAMTS4, ADAMTS9, CD44, RRM2, ***CXCL2***, CDKN1A |
| **Stress** | ***CXCL8***, IL6, MMP9, UBE2C, ***CXCL1, FPR1***, ACAN, SERPINA1, AGTR1, CDK1, COL7A1, PLAUR, VEGFA, RUNX1, VNN1, CSF2, ***BDKRB2, BDKRB1***, KIF11, SOCS3, HBEGF, ADAMTS4, ADAMTS9, EXO1, ICAM1, ***CXCL2***, AGTR2, SYT9, RRM2, CCR2 |
| **Clustering Coefficient** | PER2, BHLHE41, PRF1, GZMB, PBK, CCL19, CXCL13, CASC5, TNFAIP6, MMP10, GTSE1, CCL7, CXCL9, CXCL5, CXCL3, CTF1, LIF, CCL11, ADORA3, P2RY14, GNLY, STON2, SGIP1, COL4A4, COL4A3, PTGFR, ESCO2, PTGER3, CHI3L1, TGFB3 |
| **Most Common genes of 12 topological algorithms** | ***FPR1, BDKRB2, CXCL2, CXCL1, BDKRB1, CXCL8*** |
